# Benchmarking the research track record and level of appointment of Australian medical laboratory science academics

**DOI:** 10.1186/s12909-020-02298-9

**Published:** 2020-10-15

**Authors:** Rebecca Donkin, Kieran Broome, Libby Swanepoel

**Affiliations:** School of Health and Sport Sciences, University of the Sunshine Coast, 90 Sippy Downs Drive, Sippy Downs, Queensland 4556 Australia

**Keywords:** Medical laboratory science, Academic, Benchmarking, Universities

## Abstract

**Background:**

Benchmarking across and within universities is a common tool to evaluate performance of a program and maintain accreditation requirements. While teaching remains a primary responsibility of many academics, academic research performance is a major contributor towards career advancement and standards in the medical laboratory science profession. While anecdotal evidence suggests academics are active contributors to the evidence base of the profession, there is a high variability in research output in relation to institution and level of appointment. The aim of the study was to benchmark the research track record of Australian medical laboratory science academics and provide insight into how research productivity informs the level of appointment of academics across their career pathway.

**Methods:**

A bibliographic analysis of Australian medical laboratory science faculty websites and corresponding Scopus citation database profiles was conducted. A description of current research track record and relationships with holding a doctorate, academic appointment level, research and teaching interests, and institutional characteristics were explored. Quantitative data and frequencies were analysed using IBM SPSS version 26 to benchmark research track records by academic appointment level.

**Results:**

There were 124 academics identified from 13 universities who had a teaching and research position in an undergraduate medical laboratory science program in Australia. Academics at the level of lecturer or higher typically held a doctorate (89%). Holding a doctorate strongly influenced the number of publications. The top 20% of researchers authored around half of the overall publications. The majority of academics did not have alignment of their major research and teaching areas however, alignment had no influence on number of publications. There was, however, an inconsistent relationship between metropolitan or regional university location and the number of publications.

**Conclusion:**

Data from this study provides academics with benchmarks for the research track record required at each level of appointment. When drawing conclusions on academic progression, promotion and tenure through research track record it would be mindful to assess each on a case by case basis. Institution (metropolitan versus regional) and research interest appears to influence publication number, *h*-index and citation scores.

## Background

Research underpins medical pathology services, with the overarching requirement to provide competent medical testing within a quality system that determines the cause and nature of diseases [1]. The role of medical testing is predominantly provided by medical laboratory scientists and technicians who perform medical laboratory tests on blood, other body fluids and tissues to assist clinicians in the diagnosis, treatment and prevention of disease. Medical laboratory scientists and technicians have multi-disciplinary training or specialise in specific disciplines such as anatomical pathology, cytology, microbiology, blood transfusion, hematology, clinical biochemistry and genetics/molecular pathology while working alongside medical and other allied health professionals and administrative staff.

The academic training for medical laboratory scientists is a 3 or 4 year bachelor’s degree, whereas training for a medical technician is usually a 2 year associate degree. Acceptable programs in Australia that contain subjects relevant to pathology and meet accreditation requirements by the Australian Institute of Medical Scientists are recognised as suitable programs to provide graduates for the profession. University education is mostly provided by medical laboratory/biomedical science academics who teach general science and health subjects such as anatomy, physiology and foundations in medical science. Most academics also specialise in medical laboratory science (MLS) subjects including: clinical chemistry; hematology; medical microbiology; transfusion science (immunohematology); histopathology/diagnostic cytology and work integrated learning (pathology placement) in one or more pathology disciplines.

Benchmarking across and within universities is a common tool to evaluate performance of a program and maintain accreditation requirements. While teaching remains a primary responsibility of many academics, academic research performance is a major contributor towards career advancement and standards in the MLS profession [2, 3]. Previous literature in occupational therapy [4] and dietetics [5] in Australia have reported a high variability in research productivity between health professions. Similar findings have been reported in equivalent clinical laboratory science (CLS) academics from the United States (US) with low research productivity, reporting 36% of CLS academics not publishing a research paper or abstract over 7 years [6] and 68% of respondents to a CLS research survey reported never participating in research activities [7]. Funding and institution are likely key features that influence research productivity [8]. It is reported in the US that the top 10% of CLS academics produce almost 50% of scholarly activities [3] suggesting research productivity is influenced by the academic institutional culture, financial support and time allocated for research, not necessarily level of academic appointment.

Pathology academics in the US have made progress in obtaining qualifications above a bachelor’s degree [6] and educators in MLS often have years of experience in clinical pathology or currently hold clinical positions in pathology, frequently with a higher degree by research (Masters or PhD) [8]. However, little is known about the research productivity of Australian MLS academics. While anecdotal evidence suggests Australian MLS academics are active contributors to the evidence base of the profession with specialisations in the field of one or more pathology disciplines, there is a high variability in research output in relation to institution and level of appointment.

The aim of the study was to benchmark the research track record of Australian medical laboratory science academics and provide insight into how research productivity informs the level of appointment of academics across their career pathway.

## Methods

A bibliographic analysis of Australian medical laboratory science faculty websites and corresponding Scopus citation database profiles was conducted. A description of current research track record and relationships with holding a doctorate, academic appointment level, research and teaching interests, and institutional characteristics were explored. The methods were adapted from previous studies benchmarking the research track record and level of appointment of other Australian allied health academics [4, 5].

The data was collected in December 2019, so all academic promotion positions were complete for that year to limit error of appointment classification. Using only publicly available data, the study was provided with an exemption from human ethical approval from the author’s institutional Human Research Ethics Committee (OE20019).

Publicly available website information from Australian universities that had an undergraduate program of study in Medical Laboratory Science, Medical Science (Pathology), Science (Laboratory Medicine) or Laboratory Medicine relevant to pathology and met accreditation requirements by the Australian Institute of Medical Scientists, were included. The university webpages were reviewed to identify academics in a current teaching and research role in a pathology discipline for example, clinical chemistry, hematology, medical microbiology, or histopathology. Academics who were exclusively research, clinical only or were in a faculty/discipline outside of MLS (e.g. dentistry) but taught into an MLS subject (for example anatomy and physiology) were excluded as their research track record would likely skew the data.

The first author (RD) collated the website data as an experienced academic in the field and having in-depth knowledge of pathology subjects and programs that meet accreditation requirements. Scopus was selected as the database with a systematic scope limited to high quality journal publications to identify each academic’s research profile. Where potential discrepancies between the academic’s university webpage data and Scopus data were found, further review of the academic’s profile through publicly available webpages was investigated. The most common discrepancy was altered name, through marriage, nick name or preferred spelling. After data entry, one author (LS) independently checked data entry accuracy in 25 randomly selected records (20% of the sample size). There were four minor discrepancies which were resolved by discussion. These included a recent promotion for one academic which changed level of appointment and merging of multiple names in Scopus. Where an academic had multiple Scopus profiles, these metrics were manually merged.

### Data collection

Data collected from the academic’s public webpage included name and university of employment (subsequently removed once matched with Scopus identity), gender, location of university or primary campus site (metropolitan local population > 1 million people or regional local population < 1 million people), university program, level of appointment (Associate Lecturer-equivalent to Instructor, Lecturer, Senior Lecturer- equivalent to Assistant Professor, Associate Professor and Professor), holding a doctorate (title of Dr. or PhD qualification), major teaching interest (MLS subject/discipline) and major research interest. Website biographies were reviewed to establish major teaching and research interest although some academics had overlap or multiple interests. These were correlated with explicit research outcomes such as grant, award and publication data if available to obtain a major theme.

**Fig. 1:**
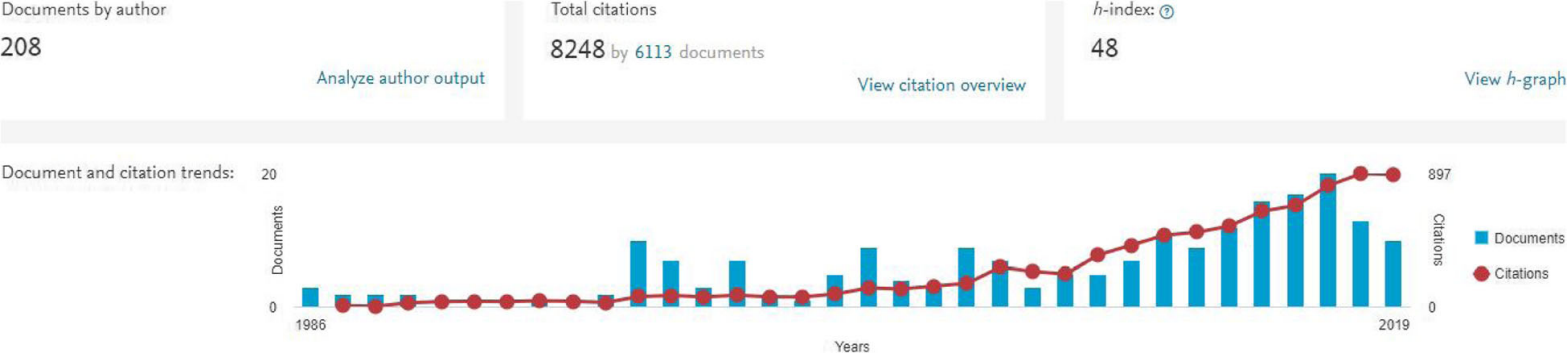
An example of publicly available Scopus documentation and citation trend for a male with an academic teaching and research appointment in medical laboratory science at level of professor with a h-index of 48

From the Scopus profile, key metrics were recorded which included: total number of documents by author; total number of citations, *h*-index, and; total number of co-authors. Scopus was chosen as the tool to measure research outputs as it automatically generates precise citation and scholarly record information by individual and institution profiles. The content is rigorously vetted and only includes articles that are indexed and reviewed by an independent review board. Unlike other services that allow the author to upload content which could produce spurious analytical outputs. The Scopus profile includes the *h-Index* which is a numerical indicator of how productive and influential a researcher is, which is more accurate than counting citation score alone to measure research impact. Research subject areas were also categorised in Scopus and were compared with the academic’s webpage information. An example of Scopus documentation and citation trends is included in Fig. 1.

### Statistical analysis

Quantitative data and frequency analytics were analysed using IBM SPSS version 26 to benchmark research track records by academic appointment level. Given the non-parametric nature of the data, medians and interquartile ranges were provided for descriptive data. Linear regression analysis was conducted to formulate an equation which would assist in predicting an academic’s level of appointment based on their research track record. Ordinal regression analysis was also conducted to confirm the linear regression analysis however, the linear regression was reported due to its ability to produce a predictive equation. Given the assumption of normality underlying linear regression analysis, non-parametric variables (i.e., Scopus data which include number of co-authors, number of publications, number of citations and *h-index*) were subject to various transformations (based on the exponential nature of these variables, logarithmic, square, cubic and quadratic roots were computed) to achieve normality, and Kolmogorov-Smirnov tests were conducted to identify the most appropriate transformation. All variables met the assumption of normality once transformed, with a logarithmic transformation most appropriate for the number of co-authors and cubic roots transformations, most appropriate for number of publications, number of citations and *h-index*.

## Results

There were 124 academics (*n* = 56 female) identified from 13 universities who had a teaching and research position in an undergraduate MLS program in Australia. Of the 13 universities with MLS undergraduate programs, five universities were considered regional (local population less than 1 million people), and eight were metropolitan (local population greater than 1 million people). The predominant academic level was senior lecturer (40%), followed by lecturer (33%), with associate professor (17%) and professor (7%) constituting less than a quarter of all academic positions. Academics at the level of lecturer or higher typically held a doctorate (89%). No academics at associate lecturer level (*n* = 3) held a doctorate and all were female.

**Fig. 2:**
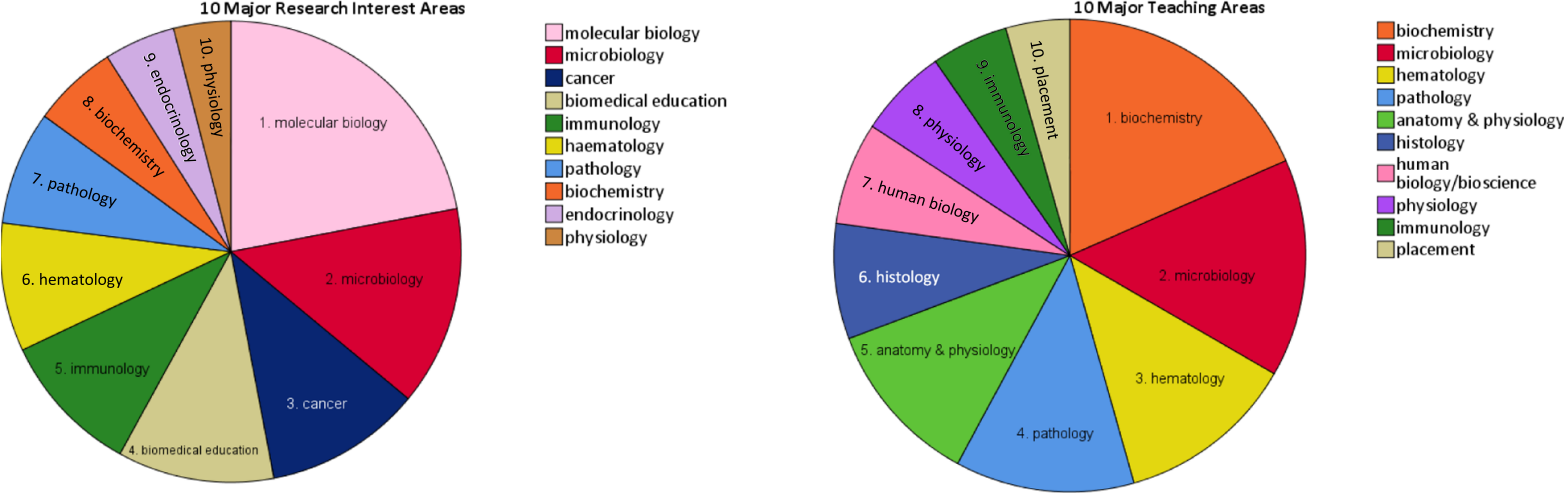
Major research interest (left) did not align with major teaching interest (right) for the majority of Australian MLS academics

The research track records of Australian academics are shown in Table 1. Associate lecturers are not shown, as no MLS associate lecturers in Australia had a research track record. The majority of academics (68.5%) did not have alignment of their major research and teaching areas, with the exception of the microbiology discipline (see Fig. 2), however alignment had no influence on number of publications (median 25 versus 27).

**Table 1:** Research track records of Australian medical laboratory science academics by level of appointment

		Academic level				
		Lecturer (*N* = 41)	Senior Lecturer (*N* = 50)	Associate Professor (*N* = 21)	Professor (*N* = 9)	All (*N* = 121)
Number of publications	Range	0–56	3–98	14–79	50–217	0–217
	Median (IQR)	10 (24)	24 (27)	53 (17)	82 (104)	26 (41)
Number of citations	Range	0–2132	33–3340	187–4492	1466–8242	0–8242
	Median (IQR)	131 (499)	448 (775)	960 (1252)	1813 (3102)	450 (995)
Number of co-authors	Range	0–174	5–585	36–2950	93–590	0–2950
	Median (IQR)	27 (64)	56.5 (63)	120 (89)	136 (219)	63 (96)
*h*-index	Range	0–24	3–26	9–25	15–48	0–48
	Median (IQR)	6 (10)	11 (9)	17 (9)	22 (14)	11 (12)

Holding a doctorate strongly influences the number of publications (median = 1 publication for those without a doctorate and 28.5 for those with a doctorate). The median number of co-authors considerably increased with academic level as did the median number of citations, which is reflected by an increase in number of publications.

**Fig. 3:**
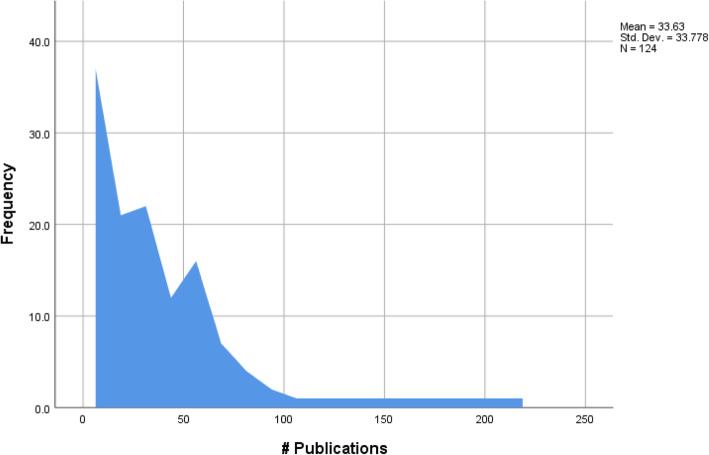
Number of publications of Australian medical laboratory science academics

Consistent with previous research [4], the number of publications was skewed towards the lower end, however this did not follow a pareto tendency (see Fig. 3). Unlike a pareto tendency, where the top 20% of researchers have authored 80% of the recorded publications, in the case of Australian MLS academics, the top 20% of researchers had authored 49% of the overall publications.

**Fig. 4:**
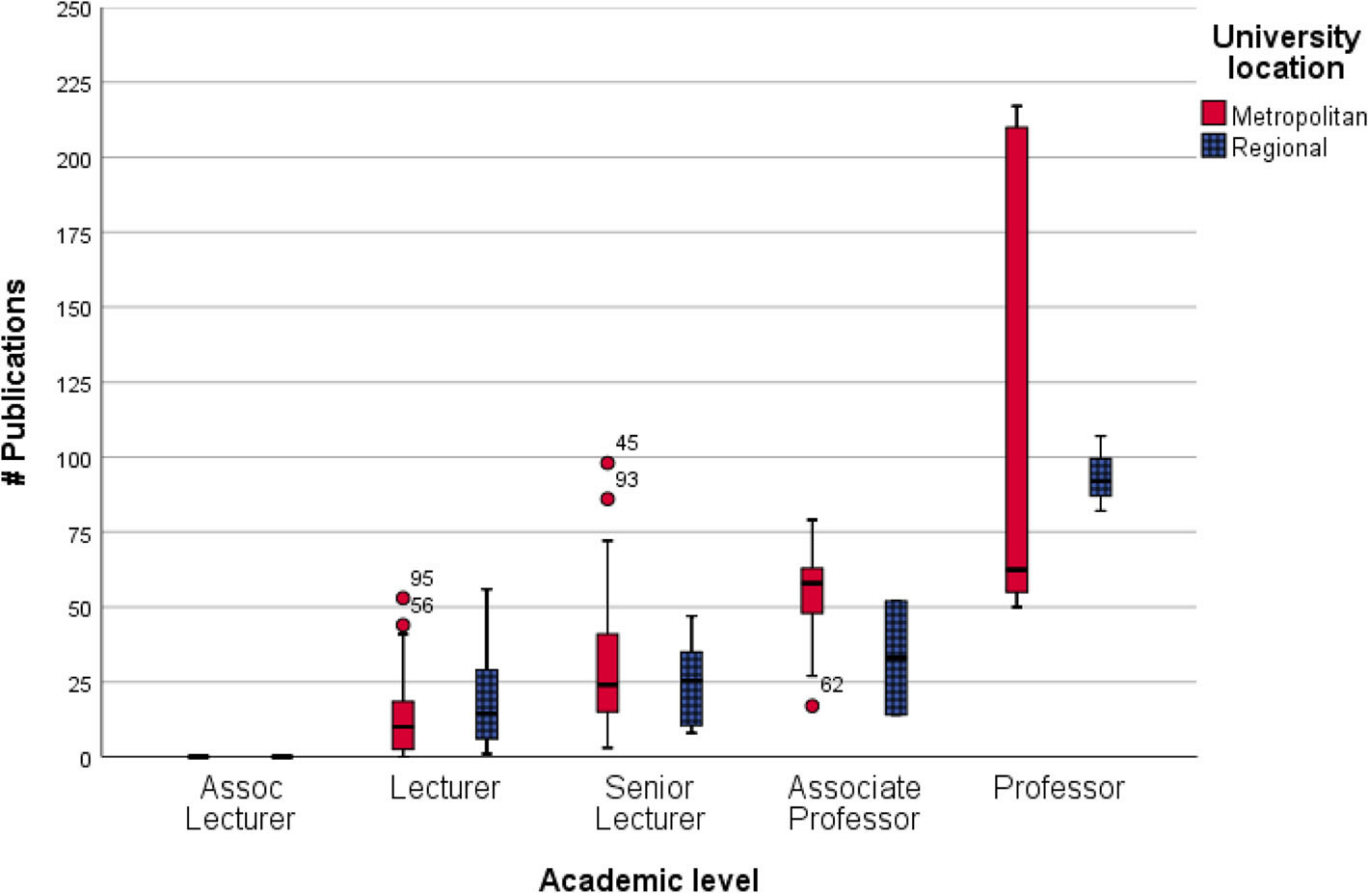
Number of publications against academic level and location of university

There was an inconsistent relationship between metropolitan or regional university location and the number of publications at each level (see Fig. 4), with lecturers tending towards a more established research track record at regional universities, and associate professors and professors tending towards a more established research track record at metropolitan universities. This may have been skewed by the small number of associate professors (*n* = 2) and professors (*n* = 3) at regional universities.

Backwards linear regression was conducted to ascertain the impact of research track record and other explanatory variables (e.g. gender) on level of appointment. Linear regression was chosen over ordinal regression for its ability to create a predictive equation for academics to benchmark their own level of appointment. A significant model (*p* < 0.001, R^2^ = 0.510) was found with only two explanatory variables; regional university location (B = − 0.288), cubic root transformation of number of publications (B = 0.572), and a constant of 0.381. Confirmatory analysis of this model was conducted using ordinal regression, which resulted in a similar model (*p* < 0.001, R^2^ = 0.576) and the same explanatory variables.

Given that a negative impact of working at a regional university on the level of appointment contrasted with the positive impact of regional employment from previous studies [4, 5] and appeared inconsistent with the need to offer employment incentives to attract quality academics to regional universities, a linear regression was also conducted without the regional university variable. This yielded similar results (R^2^ = 0.492, model significance *p* < 0.001, constant 0.260, cubic root transformation of number of publications B = 0.588).

## Discussion

Data from this study provides MLS academics with benchmarks for the research track record required at each level of appointment. A predictive model as shown below explained more than 50% of variation in level of appointment. Academics wishing to calculate their own expected level of appointment based on research track record can use the following predictive equation.

$$ \mathsf{Academic}\ \mathsf{level}\ \left(\mathsf{where}\ \mathsf{associate}\ \mathsf{lecturer}=\mathsf{0}\ \mathsf{t}\mathsf{o}\ \mathsf{professor}=\mathsf{4}\right)=\mathsf{0}.\mathsf{381}+\left(-\mathsf{0.288}\ast \mathsf{works}\ \mathsf{a}\mathsf{t}\ \mathsf{a}\ \mathsf{regional}\ \mathsf{university}\ \left[\mathsf{yes}=\mathsf{1},\mathsf{no}=\mathsf{0}\right]\right)+\left(\mathsf{0.572}\ast \mathsf{cubic}\ \mathsf{root}\ \mathsf{of}\ \mathsf{number}\ \mathsf{of}\ \mathsf{publications}\right) $$

Interestingly, working at a regional university negatively impacted on the level of appointment relative to research track record. This contrasts with the field of occupational therapy, where working in a regional university tended to elevate the level of appointment [4]. The impact of regional status on MLS academics is contrary to typical principles of providing regional incentives for employment. This is worth further exploration by the profession, as the current treatment of regional academics may be inequitable. When the influence of regional employment was removed from the linear regression model, the equation suggests that the number of publications expected for each academic level would be; Associate Lecturer 0 papers, Lecturer 0 to 9, Senior Lecturer 9 to 55, Associate Professor 55 to 167, and Professor 167 and over.

The benchmarks and predictive algorithm in this study were different to that of similar samples of Australian occupational therapy and dietetic academics [4, 5]. At the lecturer level, the median number of publications for MLS academics (10) was higher than occupational therapy (2) and dietetic (6) academics, while at the professorial level the median number of publications for MLS academics (82) was between occupational therapy (69) and dietetic (96) academics. Similar to the dietetics profession, the MLS predictive algorithm included number of publications, but no reference to reach (e.g., citations or *h*-index), which suggests that publication quantity is currently key to academic advancement. In contrast to occupational therapy, MLS academics working at a regional university had a negative rather than positive effect on level of appointment. Possible reasons for this variation may be due to greater access to research funding in MLS compared with allied health fields, or less emphasis on clinical backgrounds or practical experience and more emphasis on research background. There is a need to focus future research on investigating whether this variance is justified. Further research into these differences could also consider funding sources and amounts, as well as the diversity of collaborators and composition of research teams.

Holding a doctorate denotes a level of recognition or credibility for research in a specific field. Like other disciplines [4, 5], holding a PhD in this sample was an important milestone for establishing a research track record, confirming the use of a doctorate in benchmarking employment in a teaching and research academic position above associate lecturer. Interestingly, higher education qualifications in Australia are subject to stringent regulatory standards that require teaching academics to hold higher degree qualifications. The Australian Qualifications Framework (AQF), the national policy for regulated qualifications in Australian education and training contains the Higher Education Standards Framework 2015 [9] which includes the requirement that the learning outcomes of all higher education qualifications must be consistent with the level of the program. The standards specify that “academic teaching staff must be qualified to at least one level of qualification (AQF level or equivalent) higher than the course of study being taught, or have equivalent relevant academic or professional or practice-based experience and expertise” [10]. This means that to fulfil the AQF + 1 rule an academic teaching in a MLS bachelor’s degree must hold a qualification above bachelor’s degree such as a Masters or PhD. So not only is holding a higher degree by research (Masters or PhD) relevant for research success but is also required to meet teaching standards in Australia.

Diversity in MLS research is lacking. Large gaps still remain in knowledge and in practice of science fields, and racial/ethnic and indigenous minorities continue to have higher rates in diseases, disabilities and premature deaths than non-minorities [11]. In contrast, molecular biology research has undergone explosive growth in publications and elevated citation frequency in the past decade [12]. Editorial preference in major biomedical science journals have moved away from basic science research in preference for clinical epidemiology, clinical trials and more recently the emphasis on quality of care, medical education, health systems, and ethics [12]. This appeared to be true for academic research in MLS with molecular biology ranked the highest major research interest at 17.7% followed closely by microbiology (11.3%), education (8.9%) and cancer (8.9%) rounding out the top research interests for MLS academics. Institution was a determining factor for *h*-index with higher scores achieved at metropolitan institutes predominantly by male academics with a research interest in molecular biology. Researching popular fields may result in elevated citation rates and *h*-index, with more papers in the field offering potential citations. While education ranked in the top research interests for MLS academics the mean *h*-index of these academics was low at 3 (range 0–10), compared to research interest in molecular biology with a mean *h*-index at 15 (range 3–25) and endocrinology with a mean *h*-index at 26 (range 17–48). As citations contribute directly to a journals impact factor, this may reflect editorial preference for clinical, translational or molecular research over basic science or education related research [12]. As was observed in a US study that the top 10% of CLS academics produced almost 50% of the scholarly activities [3] in Australia 20% of MLS academics produce approximately 50% of the total publications.

Individual academics and university departments are evaluated and ranked according to research activity and performance. An accurate and reliable system of assessment and ranking, that is equitable to variances between disciplines is needed [13]. University-wide expectations of research performance are often applied to promotion without considering intricacies and variances between disciplines. For example, for academics publishing high quality research of value to society in less populous fields (e.g., medical education), it may be prudent to accept lower citation rates when judging against benchmarks. Given the variance in research productivity of MLS academics compared with other allied health academics (occupational therapy and dietetics), an understanding of benchmarks for each discipline is warranted.

### Limitations

While the *h*-index does not account for first author and has minor flaws, the *h*-index is accepted as a measure of both quality and quantity in research publications and has been used previously to evaluate academic departments [14]. Review articles can have significant citation impact and may be increasingly preferred by journals as they attempt to increase their impact factor [15]. Original research articles, in comparison to review articles, may be a better reflection of the scientific performance of an individual, however they are generally cited less frequently thus negatively influencing an individual’s citation profile [16]. Further investigation of the type of research publications would provide insight into the depth and diversity of research published in the MLS field. The authors are mindful that Scopus does not account for career break or change in career, further exploration of career journey would provide a better understanding of the influences on personal research productivity.

## Conclusions

This study benchmarks the research track record of Australian MLS academics. We developed a predictive algorithm that can be used to evaluate the research performance of individuals and university departments. This study highlights distinct variations in research track record and level of appointment of MLS academics and provides insight into how research performance informs level of appointment across the career pathway. When making decisions on academic progression, promotion and tenure, university management should be mindful of narrow measures of research productivity, we encourage them to also consider individual academic’s institutional location, research interest and contribution to teaching and learning.

## Data Availability

The datasets used and/or analysed during the current study are available from the corresponding author on reasonable request.

## Abbreviations

MLS: Medical laboratory science
CLS: Clinical laboratory science
AQF: Australian Qualifications Framework
